# Supplementary material for: The Manifold Bioactivity and Immunoreactivity of Microbial Proteins of Cow and Human Mature Milk in Late Lactation
Source: Animals (Basel). 2022 Sep 28;12(19):2605. doi: 10.3390/ani12192605 (PMC9558504; doi:10.3390/ani12192605)
Supplement: Supplementary file 1 [file animals-12-02605-s001.zip › animals-1913084-supplementary/Supplementary data 28.08.2022.pdf]

Supplementary data

Human sere description:

Human sera were collected from 6 polyallergic patients 6–17 years (average 8.4 years). Total serum IgE, estimated with ImmunoCAP (Phadia UniCAP system, Uppsala, Sweden), ranged 107-1777 kU/L. The presence of specific IgE antibodies against food antigens (including: anti cow's milk antigensess: f76: alpha-lactalbumin, f77: beta-lactoglobulin, f78: casein; anti hens egg: f75: hens egg yolk; f51-soybean; f49: apple); animal dander (against cat: e1 and dog: e5); against poolen: birch: t3; meadow grass: g8; mugwort: w6. Allegry to food, pollen as well as animal dander) was confirmed with immunoblot (Allergy Profile Pediatric test, DP 3712–1601E; EUROIMMUN, Wroclaw, Poland). Total serum IgG, estimated with IgG (Total) Human ELISA Kit (BMS2091; Thermo Fisher, Life Technologies, Poland), ranged6.8-14.5 mg/mL. No specific antibodies in control 6 sera and total IgE<16kU/mL, total IgG<4 mg/mL were confirmed. The detailed characteristics of serum was included in the authors' publication REF: Ogrodowczyk AM, Zakrzewska M, Romaszko E, Wróblewska B. Gestational Dysfunction-Driven Diets and Probiotic Supplementation Correlate with the Profile of Allergen-Specific Antibodies in the Serum of Allergy Sufferers. Nutrients. 2020 Aug 9;12(8):2381. doi: 10.3390/nu12082381. PMID: 32784846; PMCID: PMC7468854.

Table S1. Dairy proteins identified by LC-MS/MS analysis in milk samples digests

Protein Name	Accession [a]	Score [b]	Mass [c]	Matches [d]	emPAI [e]	Protein sequence coverage [f]	Host organism
**Cow's milk proteins**							
Beta-lactoglobulin	P02754	9221	19,870	420	186.95	43	*Bos taurus*
Alpha-S1-casein	P02662	7356	24,513	994	195.07	33	*Bos taurus*
Beta-casein	P02666	828	25,137	203	7.72	51	*Bos taurus*
Alpha-lactalbumin	P00711	165	16,236	7	2.59	26	*Bos taurus*
Xanthine dehydrogenase/oxidase	F1MUT3	100	148,417	11	0.09	4	*Bos taurus*
Immunoglobulin J chain	Q3SYR8	81	18,260	2	0.25	9	*Bos taurus*
Complement C3	Q2UVX4	80	188,377	4	0.05	3	*Bos taurus*
Kappa-casein	A0A140T8A9	79	21,316	10	0.22	22	*Bos taurus*
Glycosylation-dependent cell adhesion molecule 1	P80195	73	17,187	4	0.27	17	*Bos taurus*
Polymeric immunoglobulin receptor	P81265	61	83,441	4	0.16	5	*Bos taurus*
Perilipin	F1N1N6	58	49,539	2	0.09	4	*Bos taurus*
**Human milk proteins**							
Beta-casein	P05814	2394	25,412	658	7174.34	77	*Homo sapiens*
Alpha-S1-casein	P47710-2	346	21,713	45	2.16	47	*Homo sapiens*
Alpha-lactalbumin	P00709	212	16,670	12	0.65	9	*Homo sapiens*
Serum albumin	P02768	206	71,317	6	0.27	4	*Homo sapiens*
Butyrophilin subfamily 1 member A1	Q13410	166	59,383	22	0.43	22	*Homo sapiens*
Polymeric immunoglobulin receptor	P01833	162	84,197	13	0.22	14	*Homo sapiens*
Immunoglobulin heavy constant mu	A0A075B6N9	113	49,960	2	0.18	15	*Homo sapiens*
Kappa-casein	P07498	107	20,339	33	3.20	26	*Homo sapiens*
Osteopontin	P10451-5	100	33,961	25	0.64	30	*Homo sapiens*
Platelet glycoprotein IIIb = CD36 antigen	E9PLT1	95	45,072	2	0.10	5	*Homo sapiens*
Immunoglobulin mu heavy chain	P0DOX6	87	64,244	2	0.37	2	*Homo sapiens*
Immunoglobulin kappa constant	A0A075B6H6	70	11,851	2	0.41	8	*Homo sapiens*
Alpha-1-antichymotrypsin	P01011	67	47,758	5	0.09	10	*Homo sapiens*
Apolipoprotein A1	Q9Y355	64	7429	2	0.72	10	*Homo sapiens*
Fatty acid synthase	A0A0U1RQF0	61	27,5142	10	0.03	4	*Homo sapiens*
Lactotransferrin	E7EQB2	59	78,094	26	0.11	13	*Homo sapiens*
Alpha-1-antitrypsin	A0A024R6I7	56	46,817	5	0.09	11	*Homo sapiens*

a UniProt/Trembl database accession number.
b The protein score derived from the ions scores of MS/MS report based on the calculated probability, when the significance threshold was chosen to be 0.05, score cut-off-43.
c Monoisotopic mass
d Number of significant peptide matches.
e The Exponentially Modified Protein Abundance Index (emPAI)
f Coverage expressed in % number of amino acids in a specific protein sequence that were found in significant peptide matches.

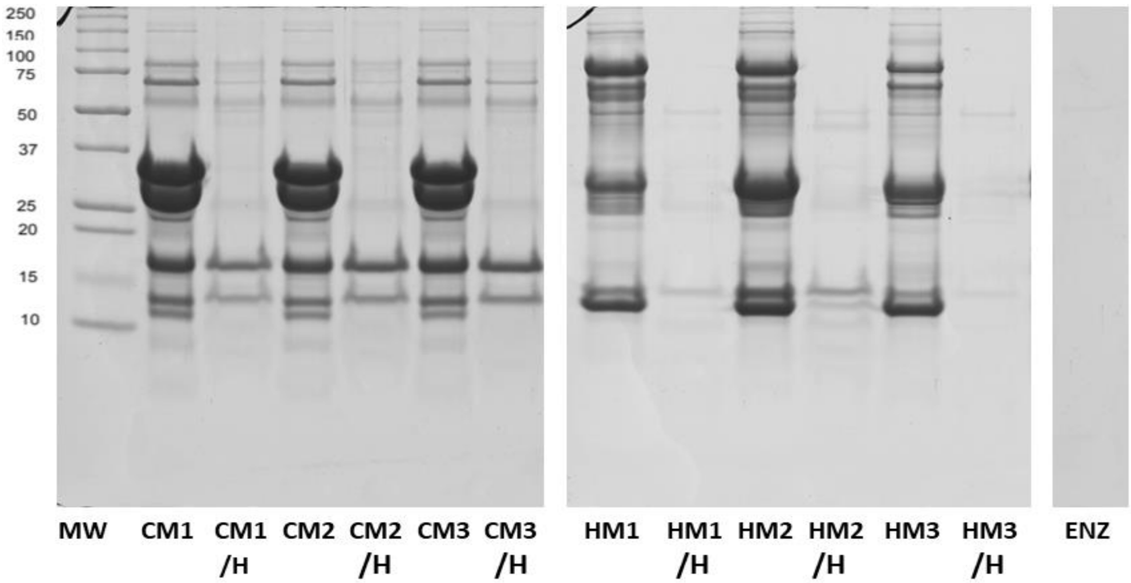

Figure S1. Electrophoretic separation of milk proteins before (CM1-3, HM1-3) and after (CM1-3/H, HM1-3/H) simulated digestion. ENZ-mixture of enzymes applied for digestion.

Table S2. *In silico* modeling of dairy proteins activity

Protein Name	Accession number [a]	Allergenicity [b]	Proinflamatory response [c]	Immunoglobulin induction [d]	IL10 inducers [e]	Bioactivity [f]							Proinflamatory response/ SVM score	IFN Inducers [c]	IL4 Inducers [d]
						anti-inflammatory	antioxidative	antibacterial	immuno-modulating	immuno-stimulating	regulating	neuropeptides			
**Cow's milk proteins**															
Beta-lactoglobulin	P02754	**Bos d 5 (164)**; E<1e-7; score: 2.0	92/1.44	IgE-1.468	1.29	-	0.270	0.033	0.006	-	0.023	0.028	92/1.44	39	125
Alpha-S1-casein	P02662	**Bos d 8 (167)**; E<1e-7; score: 2.0	120/1.38	IgE-1.473	1.50	-	0.224	0.083	0.009	0.009	0.005	0.014	120/1.38	44	142
Beta-casein	P02666	**Bos d 11.0101 (10199)**; E<1e-7; score: 2.0	130/1.31	IgE-1.371; IgA-1.488	1.19	0.013	0.143	0.033	0.027	0.005	0.049	0.005	130/1.31	42	133
Alpha-lactalbumin	P00711	**Bos d 4 (163)**; E<1e-7; score: 2.0	56/1.29	IgE-1.286; IgA-0.975	1.14	0.007	0.070	0.033	0.028	-	0.021	-	56/1.29	28	50
Xanthine dehydrogenase/oxidase	F1MUT3	no matches	567/1.67	IgG-1.724; IgA-0.957	1.23	0.004	0.068	-	0.005	0.001	0.016	0.008	567/1.67	ND	ND
Immunoglobulin J chain	Q3SYR8	no matches	47/1.31	IgG-0.955; IgA-1.010	1.35	-	0.051	-	-	-	-	0.013	47/1.31	20	124
Complement C3	Q2UVX4	no matches	716/1.59	IgG-1.665; IgE- 0.952; IgA-1.197	1.24	-	0.088	-	0.003	0.002	0.014	0.014	716/1.59	ND	ND
Kappa-casein	A0A140T8A9	**Bos d 12 (2737)**; E<1e-7; score: 1.92	78/1.18	IgE-1.272	0.92	0.021	0.168	0.067	0.005	-	-	-	78/1.18	36	131
Glycosylation-dependent cell adhesion molecule 1	P80195	21.7% identity (51.4% similar) with allergen Lep s 1; E=0.99	50/1.34	IgG-1.100	1.15	0.007	0.065	-	-	-	0.020	0.007	50/1.34	26	116
Polymeric immunoglobulin receptor	P81265	35.4% identity (57.3% similar) with allergen Bos d 13; E= 0.44; score: 0.43	286/1.67	IgG-1.621; IgE-1.497; IgA-1.049	0.99	0.008	0.052	-	0.003	-	0.023	0.001	286/1.67	ND	ND
Alpha-2-HS-glycoprotein	B0JYN6	32.0% identity with auto-allergen Hom s TPA; E= 0.64; score: 0.42	121/1.36	IgG-1.220; IgE-0.953; IgA-1.154	1.15	0.003	0.061	-	0.003	0.003	0.020	0.008	121/1.36	56	236
Perilipin	F1N1N6	34.4% identity (55.6% similar) with allergen **Bos d 13**; E= 0.6	201/1.35	IgG-1.111 ; IgE-1.284 ; IgA-1.195	1.21	0.002	0.060	-	-	0.002	0.016	0.016	201/1.35	82	291
**Human milk proteins**															
Beta-casein	P05814	**Hom s 8 (1064)**;E<1e-7; 1.84 and 56.5% identity (74.0% similar) with allergen **Bos d 11**; E<1e-7	78/1.15	IgE-1.199 ; IgA-1.219	1.15	0.006	0.117	-	0.006	0.011	0.011	-	78/1.15	11	106
Alpha-S1-casein	P47710-2	37.6% identity (57.0% similar) with allergen **Bos d 9; E<1e-7;** score: 0.73	64/1.29	IgG-0.904; IgE-1.023	1.19	0.005	0.081	-	-	-	0.005	-	64/1.29	20	93
Alpha-lactalbumin	P00709	**Hom s ALA (1289)**;E<1e-7; 1.8 and 73.9% identity (89.4% similar)with allergen **Bos d 4**; E<1e-7	60/1.22	IgG-1.126	1.22	-	0.083	-	0.033	-	-	-	60/1.22	22	72
Serum albumin	A0A087WWT3	49.6% identity (60.5% similar) with allergen **Bos d 6**; E<1e-7	156/1.8	IgG-1.189; IgA-1.248	1.24	-	0.083	-	-	-	0.017	0.017			
Butyrophilin subfamily 1 member A1	Q13410	no matches	206/1.53	IgG-1.489 ;	1.33	0.006	0.077	-	0.004	0.002	0.017	0.004	206/1.53	112	237
Polymeric immunoglobulin receptor	P01833	no matches	318/1.68	IgG-1.266; IgA-0.986	1.11	0.008	0.050	-	0.001	0.003	0.020	0.008	318/1.68	285	83
Immunoglobulin heavy constant mu	A0A075B6N9	no matches	107/1.53	IgG-1.244; IgE-0.988; IgA-0.991	1.36	-	0.017	-	-	-	-	-	107/1.53	103	253
Kappa-casein	P07498	53.2% identity (71.5% similar) with allergen **Bos d 12**; E<1e-7; score: 1.46	66/1.44	IgA-0.967	0.95	0.050	0.017	-	0.017	-	-	-	66/1.44	49	59
Osteopontin	P10451-5	25.2% identity (55.3% similar) with allergen Gly m 6.0401; E=0.9	128/1.3	IgG-1.306; IgA-0.907	0.97	-	0.050	-	-	-	-	-	128/1.3	61	195
Platelet glycoprotein IIIb = CD36 antigen	E9PLT1	no matches	186/1.81	IgG-1.411; IgA-1.303	1.36	0.017	0.050	-	0.017	-	0.033	0.017	186/1.81	31	270
Immunoglobulin heavy variable 3-33	A0A0B4J1V3	no matches	46/1.42	-	1.36	-	0.017	-	0.017	-	0.033	0.017	46/1.42	26	53
Immunoglobulin kappa constant	A0A075B6H6	**Hom s HLA-DR-alpha (8362)**; E=0.015; score: 0.78	38/1.38	IgG-1.142	1.27	-	0.033	-	-	-	-	-	38/1.38	5	37
Alpha-1-antichymotrypsin	P01011	30.3% identity (69.2% similar) with allergen Gal d 20.58; E<1e-7; score: 0.54	278/1.59	IgG-1.301; IgA-0.946	1.59	-	0.033	-	-	-	0.017	-	278/1.59	51	168
Apolipoprotein A1	Q9Y355	40.9% identity (63.6% similar) with allergen Lol p 11; E=0.41	18/1.62	IgG- 1.010	1.40	0.017	0.083	-	-	-	-	-	18/1.62	15	15
Fatty acid synthase	A0A0U1RQF0	no matches	1147/1.77	IgG-1.453; IgE-1.325; IgA-1.110	1.36	0.017	0.067	-	0.017	-	0.017	-	1147/1.77	ND	ND
Lactotransferrin	E7EQB2	**Hom s LF (1384)**; E<1e-7 and 69.9% identity (89.2% similar) with allergen **Bos d LF;** E<1e-7; score: 1.09	330/1.46	IgG-1.090; IgA-1.286	1.30	-	0.017	0.050	-	-	0.017	-	330/1.46	ND	ND
Alpha-1-antitrypsin	A0A024R6I7	30.3% identity (65.7% similar) with allergen Gal d 2.0101; E<1e-7; score: 1.66	201/1.72	IgG-1.477; IgA-1.239	1.21	-	0.067	-	-	-	0.017	-	201/1.72	37	210

[a] UniProt/Trembl database accession number.

[b] Allergenicity of protein estimated using Allergen Online database (http://www.allergenonline.org) using the Full FASTA 36 search algorithm (E-value Cutoff=1) and with Allergome database (http://www.allergome.org) using the NCBI blastp algorithm with E-value Cutoff=1; (cross-reactivity is not likely for proteins with less than 50% identity and similarity and E<1e-4 but for >70% over the entire protein sequence, and E<1e-7 is likely). It was also provided the Hybrid score for the protein with the identity to the allergenic sequence based on AlgPred 2.0 web server (https://webs.iiitd.edu.in/raghava/algpred/).

[c] Hypothetical proinflammatory activity of proteins predicted based on browser ProInflam (http://metagenomics.iiserb.ac.in/proinflam/) with default settings window length: 20 and threshold values: 0.7. Values presented as number of peptides showing inducer potential/ score for the strongest inducer.

[d] The score value of potential of antibody-specific B-cell epitopes estimated using IgPred web server (https://webs.iiitd.edu.in/raghava/igpred/) with default settings window length: 20 and threshold values: 0.9 which scan a protein to identify IgG-, IgA- or IgE-specific B-cell epitopes.

[e] Hypothetical regulatory activity through ability to induce secretion of IL-10 tested with IL10Pred tool (https://webs.iiitd.edu.in/raghava/il10pred/) with default settings window length: 9 and threshold values: 0.7. Values presented as a score for the strongest inducer.

[f] Bioactivity of proteins was tested with BIOPEP tool (https://biochemia.uwm.edu.pl/biopep-uwm/). Results presented as the A score calculated as the frequency of bioactive fragments occurrence in a protein sequence.

ND – not available data due to the database too long >500AA protein sequence /or peptide sequence was too short and did not meet the criterion of min. 9AA.
